# A novel characterisation protocol of mechanical interactions between the ground and a tibial prosthesis for long jump

**DOI:** 10.1038/s41598-023-31981-2

**Published:** 2023-03-30

**Authors:** Élodie Doyen, Fabien Szmytka, Jean-François Semblat

**Affiliations:** IMSIA, CEA, CNRS, EDF, ENSTA Paris, Institut Polytechnique de Paris, 91120 Palaiseau, France

**Keywords:** Mechanical engineering, Characterization and analytical techniques, Engineering, Materials science

## Abstract

The mechanical study of Running Specific Prostheses (RSPs) is often limited to the blade. The setup developed and presented herein is a simple experiment, based on a mechanical testing machine and a camera, that assesses two indicators relevant to coaches and athletes in the field of athletics: secant stiffness and energy dissipation. The influence of four parameters on global prosthesis behaviour is evaluated: the load line offset, the prosthesis-ground angle, the sole type and the flooring type. The load line offset and the flooring type have little to no influence on their behaviour. The prosthesis-ground angle impacts the stiffness: an increase in the angle brings a significant decrease in stiffness, which strongly impacts the performance. The type of sole modifies the kinematics of the blade tip’s interaction with the ground. However, this effect is less likely to enhance the sports practice since athletics imposes the use of spikes. The camera images allow assessing the local behaviour of the sole, thus enabling to follow its strain through the compression process.

## Athletics prostheses characterisation

Despite the difficulty of finding accurate data on the number of amputees, a recent study^1^ estimates that 65 million people live with limb amputation over the world due to traumatic causes and cancer only. Among them, at least 35.3 million have lower limb amputation that necessitates the use of a prosthetic device. Another study^2^ estimated that in 2012, within the lower limb amputations, 60% have a transtibial amputation, representing around 21.2 million people. All these people need their prostheses for all activities of daily life.

In the last decades, improvements in daily life prostheses design have enabled most of these people to find back a normal life after their amputation. Moreover, the advances in the composite material field in the 1980s greatly improved the sport-specific prostheses design and enabled more amputees to access sport^3^ and improve their performances.

The prostheses used in athletics for lower limb amputation are called Running Specific Prosthesis (RSPs). Extensive research is dedicated to them, ranging from the difference in sporting gestures between the level of amputation^4^ or between Olympic and Paralympic athletes^5^ to the behaviour of the prostheses and the interaction between the prosthesis and the limb^6^.

**Figure 1:**
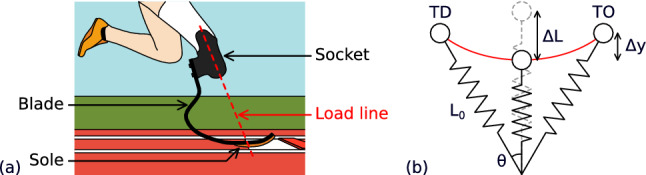
(**a**) The various RSPs components and the load line (extension of the tibia axis). (**b**) Spring-mass model used in the literature for leg and/or prosthesis stiffness (TD for touchdown of the stride and TO for take-off).

Having a closer look at tibial RSPs (amputation under the knee level), we can define three different parts: the socket, the blade, and the sole (see Fig. 1a). The first one enables the link with the athlete’s limb, the second one stores and releases energy to move forward, and the last one makes the links with the ground. The blade is the main body of the RSPs and is nowadays made of a single piece of carbon fibre that acts like a spring (see Fig. 1b). The sole rules the grip on the ground and the rotation of the blade around the contact point. Since no hinge plays the role of the ankle, only the shape of the blade tip combined with the sole allows the athlete to move forward.

The socket is totally personal and well studied by medical and biomechanical teams for at least 20 years^7^. The main issue in its design is to find the shape suited for each athlete or patient^8,9^ to guarantee comfort and avoid the risk of injury. For that purpose, the pressure repartition on the stump is usually studied and different sensors or methods are developed to optimise the socket shape and materials^10–12^.

To choose the most suitable blade for an athlete for performance, manufacturers propose recommended stiffness categories according to the body mass and the distance of the run (long-distance or sprint). However, this choice remains a suggestion of the manufacturer since it strongly depends on the level of practice and all the other parameters that the athletes can adjust by themselves. For instance, the load line (see Fig. 1a) is a parameter defined by prosthetists. They use it to position the blade with respect to the socket. The load line corresponds to the tibia axis and is meant to intersect a specific point at the tip of the blade. This setting depends on the blade shape and is specified by the manufacturer. It is given to help the athlete’s gait to be balanced with their good leg. But the load line is also a performance parameter that defines the area of the blade in contact with the ground at rest and the athletes can tune it to try to improve their performances.

However, the literature mainly focuses on the blade stiffness and various studies can be found on that subject while other prosthesis parameters are neglected. Many studies involve modelling the behaviour of the blade to better represent the experimental results. They use parametric modelling^13,14^, finite element modelling^15^ or biomechanical models^16^. The simplest modelling for RSPs is the same as the one used for modelling legs during the running or walking stance phase: namely the spring-mass model^17,18^. Both legs and prostheses are assumed to behave like a spring during the stance phase of walking, running, and jumping. They accumulate energy between heel strike (touchdown) and mid-stance and restore it from mid-stance to take-off (see Fig. 1b). On the other side, research teams develop wearable sensors^19,20^ to be mounted directly on the blade during sports practice for a better in situ assessment and optimal prescription to the user. Other teams study the blade’s stiffness in the laboratory, in quasi-static or dynamic conditions. In quasi-statics, for example, Beck et al.^21^ demonstrated that the prosthesis’s stiffness is better described by a quadratic function rather than a linear one. They also showed that the stiffness categories do not correspond to the measured stiffness of the blade and that it varies with the testing conditions—such as the angle between the longitudinal axis of the RSPs and the direction of the applied force—or between prosthetic models (shape and manufacturers). In dynamics, Petrone et al.^22^ designed a setup for the static and dynamic characterisation of RSPs reproducing the complete kinematics of a stride with different actuators. They confirmed the results of Beck et al. showing that the stiffness depends on the prosthesis-ground angle as well as the alignment of the load line.

Then the sole of the prosthesis is not studied at all to our knowledge. However, literature overflow with studies on normal shoe soles for better performance^23^, better understanding of kinematics^24^, or limitation of injury^25,26^.

More specifically, the interactions (contact, dissipated energy) between the prosthesis and the ground on which the athlete moves are hardly studied in the literature. Thus, the objective of this study is to develop an experimental protocol that will make it possible to better estimate the role of various parameters of the prosthesis (load line adjustment, nature of the sole) as well as the nature of the ground on two mechanical quantities which play a major role in the intrinsic performance of the prosthesis used by the athlete. The first is the apparent stiffness of the prosthesis at the time of its compression after contact with the ground. This stiffness determines the power of the athlete’s rebound during the long jump, but if it is too high, it can also be a source of injury. The second is the energy dissipated at each contact with the ground. It is also measured because, if too large, it can degrade performance. The role of the sporting gesture through the contact angle between the prosthesis and the ground is also investigated.

To achieve this, an original experimental setup is proposed in this study. It is based, first of all, on a mechanical test bench, available in most mechanical laboratories, so that it can be easily deployed. Only the design of specific adapters for the prosthesis needs to be considered: a first one to keep it in the loading axis of the machine, and a second one to adapt the contact angle. Finally, a camera is added to measure the local deformation of the sole by image correlation. This quantity is then analysed and its influence on the performance discussed.

## Investigating the behaviour of running specific prostheses

### Innovative setup for assessing the quasi-static behaviour

**Figure 2:**
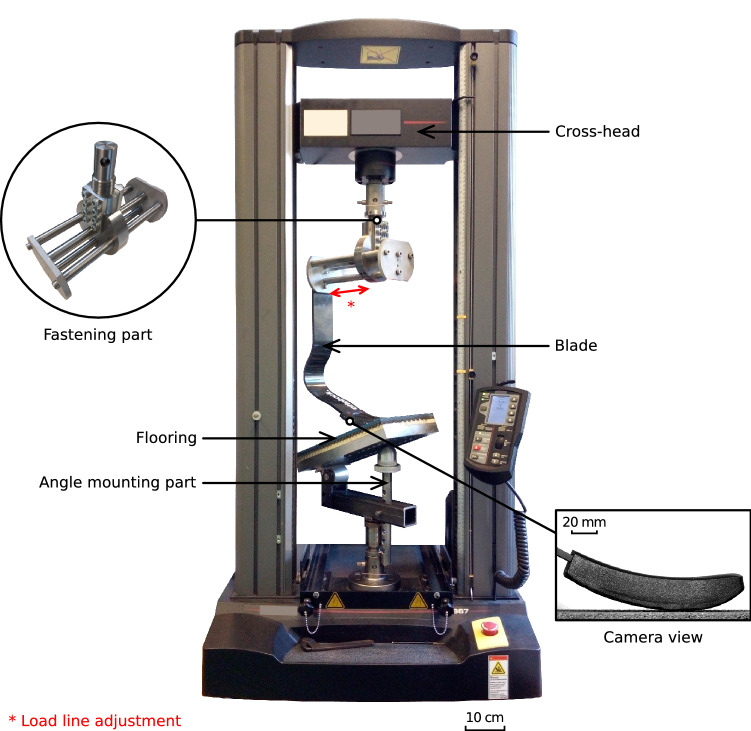
Setup to assess the stiffness and behaviour of RSPs in uniaxial compression at low speed. The fastening and angle mounting part allow the parameters of each test to be adjusted; the flooring and the sole are interchangeable as well. The blade is fixed directly to the fastening system by two screws. The sole is filmed by a camera positioned in front of its profile. The cross-head imposes the compression movement on the blade.

As previously stated, there is a lack of studies for estimating the influences of RSPs setting and usage (athlete’s gesture) on the RSPs/ground interaction. Based on standard laboratory equipment and a dedicated and original mounting part, a new setup is proposed to investigate the interaction between the prosthesis and the flooring. It is detailed in Fig. 2. A 45$^\circ$ rotation with respect to the machine frame is required to accommodate the large mount and blade, while still allowing the experiment and particularly the interaction between the sole and the floor to be filmed.

The socket is removed from the prosthesis, only keeping the blade and the sole. The blade is fastened at the top to an electromechanical testing machine with the same fastening system as when attached to the socket. The specific mounting part is detailed in the following. During the tests, a camera records the profile of the sole in close-up.

The electromechanical testing machine (Instron 5967) is mounted with a 30 kN load cell (2580 Series static; class 0.5). For this study a calibration is performed for the force cell and shows a maximum error of 0.0019% in the measured force range. This corresponds to a maximum relative error of 4 N on the force measurement, which is neglected in the remainder of the study.

The top of the blade is fastened to the cross-head of the testing machine thanks to an innovative assembly part. This part is designed to move horizontally and holds the alignment of the load line at different locations. It consists of four 12 mm diameter stainless steel bars. Three of them guide the translation to adjust the load line alignment and the last one allows to block the translation thanks to two screws to keep the desired position. The design makes the part both rigid to avoid warping of the assembly and compact to avoid taking up valuable space in the testing machine.

The lower part of the assembly is a horizontal plane where different types of flooring can be placed. It is composed of three parts screwed or fastened between them for stability and reproducibility. First, a compression plate is placed in the bottom mount of the machine to ensure horizontality, then an aluminium plate of 200 $\times$ 350 $\times$ 25 mm is placed on it to ensure that the blade always presses on a rigid ground, and lastly a flooring part covers it to reproduce real athletic surfaces. These flooring parts are detailed in the following.

A camera is positioned in the axis of the test to record the contact between the sole and the ground. The field of view is centred on the sole, to measure, as explained in the following section, its deformations.The camera has a 12 Mpx resolution and is set at a 5 Hz frame rate (Dantec Dynamics, Q-400 system camera). The lens is high resolution optimised for machine vision (Schneider Kreuznach Germany, included in the Q-400 system by Dantec Dynamics). For the processing, the image is reduced to a useful area of 2.452 $\times$ 1.226 px thus the resolution is 15 $\hbox {px\,mm}^{-1}$.

During a test, the blade is compressed vertically on the bottom part by the cross-head of the testing machine. The load is applied by a prescribed displacement. It is important to point out that this choice, instead of a force control, may be surprising compared to the sporting gesture performed in reality. Indeed, the athlete applies a force to deform the blade of her/his prosthesis. This effort is even sometimes sufficient to break the carbon blade. The blade used in this study does not allow for efforts as high as those recorded in the world’s best performers. To avoid the risk of breaking the blade during testing, displacement control seems to be the least hazardous choice. However, the setup allows for force control testing. A loading of 50 mm followed by unloading of 50 mm, both at a 1 $\hbox {mm\,s}^{-1}$ rate, is applied. The displacement of 50 mm is here small enough not to risk damaging the blade and large enough to have time to assess the behaviour during the loading/unloading process. The 1 $\hbox {mm\,s}^{-1}$ loading rate is in the fastest range the testing machine can safely reach; it is much slower than a real stride, but that speed let the characterisation of the different investigated blade properties possible.

In the next section, we describe the four adjustable parameters of the setup to assess the mechanical behaviour of prostheses: the alignment of the load line, the prosthesis-ground angle, and the types of flooring and sole.

### Blade specifications and experimental parameters

The setup can assess the behaviour of different J-shaped blades^3^. The J-shaped blades consist of a vertical part and are linked to the socket side by screws. Characterising the behaviour of C-shaped blades would be possible by changing the upper part of the mount. Results in the present works are presented for an Ottobock 1E90 Sprinter blade (see the blade in Fig. 2).

#### Load line alignment

**Figure 3:**
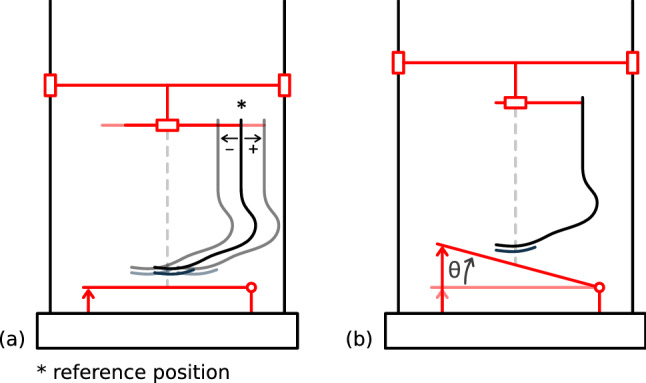
The adjustable geometric parameters. (**a**) The load line alignment with the positive and negative shifting. (**b**) The prosthesis-ground angle with respect to the horizontal.

As mentioned before, the load line is adjustable thanks to the upper mounting part. The alignment suggested by prosthesis manufacturers is considered as the reference position; the blade is positioned thanks to the mark on the sole supplied with it and a plumb line aligned with the load axis of the machine. Then the position of the vertical part of the blade is moved forward or backwards with respect to the reference position to change the load line in a ${\pm 40}\,\hbox {mm}$. A positive value indicates that the vertical part of the blade is moved away (backwards) from the load axis of the machine and a negative value indicates that it is moved toward it (see Fig. 3a).

#### Prosthesis-ground angle

The ground default position is horizontal, taken as $\theta =$ 0$^\circ$ but the setup enables tilting it to better represent the real angle of contact with the ground (see $\theta$ in Fig. 3b). The different angles are chosen from the study of high-speed images of amputees practising long jump. The angle between the ground and the straight part of the blade is measured during the last stride from touchdown to take-off. The angles range from ${-22.5\pm 2.5}^\circ$ at touchdown to ${37.5\pm 2.5}^\circ$ at take-off. However, the negative angles of touchdown are not reachable by the setup, thus the minimum angle is chosen at 0$^\circ$. At mid-stance (half of the stride in time) the angle lies between 20$^\circ$ and 25$^\circ$. Consequently, we decided to study the blade behaviour at $0^\circ$, $10^\circ$, $20^\circ$ and $30^\circ$ in first approach.

#### Sole type

Different kinds of soles can be mounted on the blade. Three different configurations are used in the present works: a running sole, a spiked sole and no sole. The running and spiked soles are the ones supplied with the 1E90 Sprinter blade in the Ottobock catalogue. The spiked sole is equipped with six 6 mm track spikes.

The side of each sole is covered with a uniform speckle pattern to monitor its strain by Digital Image Correlation (DIC). The speckle is made of white matt paint spots, of random sizes, on a uniform black matt background. Both layers have been applied by aerosol spray.

#### Flooring type

Different kinds of flooring can be mounted on the lower part of the mounting. Six types of flooring are considered: two take-off boards (TOB) and four athletics tracks (AT). The athletics tracks are provided by MONDO, the take-off boards are manufactured by Dimasport and are certified by World Athletics for competitions.

**Figure 4:**
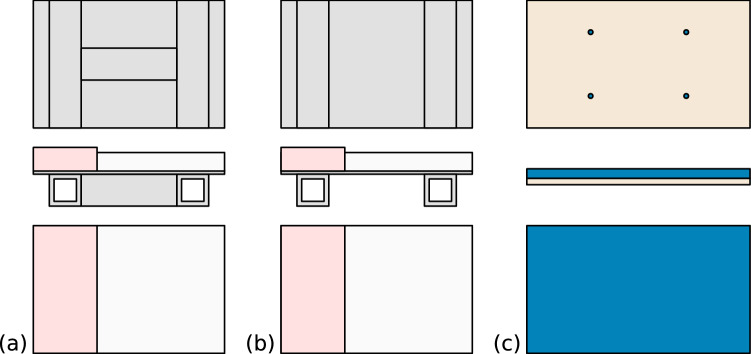
Schematics of the different flooring parts. (**a**,**b**) The two different take-off boards. Under the board, composed of a wooden plank and a thin aluminium plate, an aluminium square bar reinforces it following its edge and, in the centre, the same type of square bar is also added. The first piece, cut in the centre of the board, has three reinforcement bars (TOB-3) and the second, cut next to the first, only two (TOB-2). (**c**) The athletics tracks with the wooden boards that support them.

The take-off board and the athletics tracks are cut to fit the aluminium plate size on the bottom mounting part. By cutting the take-off board, two types of parts appeared due to the metal frame under the wooden plate (see Fig. 4).

For the athletic track, the pieces are glued to a wooden plate with the same adhesive used to bound the track on the ground in stadiums. This is a bi-component glue provided by MONDO (epoxy-polyurethane glue PU300). The thickness, weight and certifications of each athletics track are summarised in Table 1.

**Table 1:** The different athletics tracks used and their specificities.

Athletics track	Reference	Thickness	Surface density	Certification
Sportflex Super X 720 K37	AT-K37	12 mm	10.5 $\hbox {kg\,m}^{-2}$	
Sportflex Super X 720 K39	AT-K39	13.5 mm	12 $\hbox {kg\,m}^{-2}$	WA product certificate
Sportflex Super X 720 K41	AT-K41	15 mm	13.5 $\hbox {kg\,m}^{-2}$	
Mondotrack WS	AT-WS	13.5 mm	12.6 $\hbox {kg\,m}^{-2}$	WA product certificate

## Identification of prosthesis mechanical properties

To characterise the behaviour of the prosthesis depending on the different parameters mentioned in the previous section, the load as a function of displacement is recorded. For each test, a maximum preload of 10 N is applied to the prosthesis. The prosthesis is moved by the crossbar, controlled by displacement (at 0.1 $\hbox {mm\,s}^{-1}$) until contact is made with the floor in use. Once the value of 10 N is reached, the position of the prosthesis is frozen while the force and displacement sensors are set to 0. The measurements presented throughout the study therefore do not show this slight preload. Each measurement with a set of parameters is done three times successively without pausing between them. The mean value of the load over the three tests is used. From these data, we identify the relevant parameters to be computed to describe the behaviour of the prosthesis. Two viscoelastic properties are used: stiffness and hysteretic dissipation. The viscoelastic behaviour of the prosthesis comes from two elements: on the one hand the material of the sole and on the other hand the curved shape and the reduction of the section of the blade. Indeed, the materials of soles are elastomers which have intrinsic viscoelastic properties and damping that should be accounted for in the behaviour of the blade. The computation of these properties is described in the following.

Then the behaviour of the sole is also investigated thanks to the images recorded by the camera. The strains are computed as detailed in the following.

### Viscoelastic properties

#### Apparent stiffness

As previously stated, manufacturers of blades use categories to prescribe prostheses fitted for athletes. These categories are assumed to reflect the stiffness of the blade. Here, displacement and forces time evolution are recorded during the test, and the stiffness is computed from these data. However, the force-displacement curves obtained are not linear, the load increase according to a polynomial curve, with a hysteresis appearing during unloading. For simplification a purely elastic model (Fig. 1b) is used for the stiffness assessment, thus a secant stiffness called *k* is computed. It is defined as the ratio of maximum recorded force $F_{max}$ to the maximum displacement $U_{max}$:

1 $$\begin{aligned} k = \displaystyle \frac{F_{max}}{U_{max}} \end{aligned}$$

This secant stiffness is used in the following as a comparative value and represents the apparent stiffness of the blade under the test conditions.

#### Hysteretic dissipation

The hysteresis in the force–displacement curve brings hysteretic dissipation. To evaluate the dissipation, we compute a damping parameter $\eta$ as the ratio of the area of the loop between loading and unloading curves and the area under a linear curve representing a perfectly linear elastic behaviour with stiffness equal to the secant stiffness computed by Eq. (1). The result is multiplied by 100 to give a percentage:

$$\begin{aligned} \eta&= \frac{\int _{0}^{U_{max}} F(x) \, \textrm{d}x - \int _{U_{max}}^{0} F(x) \, \textrm{d}x}{\int _{0}^{U_{max}} \frac{F_{max}}{U_{max}} x \, \textrm{d}x }\times 100 = \frac{\int _{0}^{U_{max}} F(x) \, \textrm{d}x - \int _{U_{max}}^{0} F(x) \, \textrm{d}x}{\frac{1}{2}kU_{max}^2}\times 100 \end{aligned}$$

**Figure 5:**
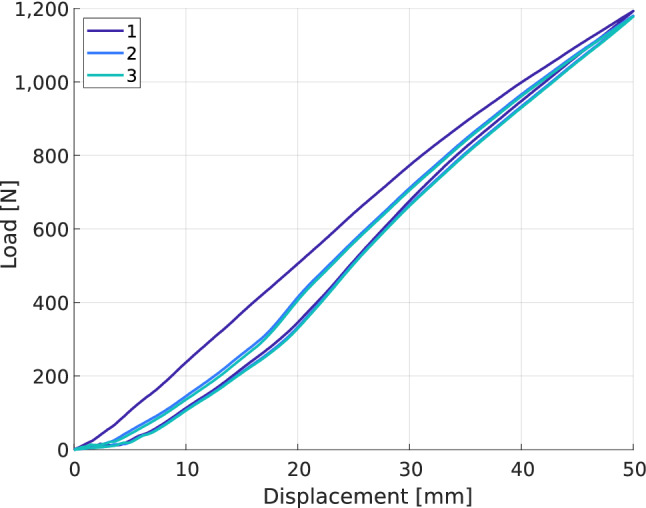
Influence of repetitive loading at the same location with the spiked sole on take-off boards: force-displacement curves of three loadings. Flooring type: TOB-2, prosthesis-ground angle: horizontal, sole type: spiked, alignment: fitted.

In configurations where the spiked sole is tested on a take-off board, the behaviour during the first loading and the two others is very different. The three successive measures are performed at the same place on the board as for the running sole, but spikes leave marks in the wood. Thus, the first measurement records the evolution of force while the spikes create the mark in the wood, whereas the two following measurements record the evolution of force for a spike entering a pre-existing mark (see Fig. 5). The resulting difference in stiffness is not significant: the stiffness of the first load cycle is 23.9 $\hbox {kN\,m}^{-1}$ while the average over the other two is 23.6 $\hbox {kN\,m}^{-1}$, i.e. a difference of 1.3%. On the other hand, the difference in dissipation is significant: on the first load cycle, it is 15.1% while it is 6.5% on average over the next two, i.e. a difference of 57%. This difference stems from the spikes taking longer to meet the full resistance of the take-off board when they sink into a pre-existing mark.

Moreover, disregarding the first load data in the configurations with the spiked sole on take-off boards globally makes the average standard deviation, computed over only two measurements, similar to that of all running sole configurations.

The reality of practice for the long jump is in between the two situations: the take-off boards always have some pre-existing marks on them due to former attempts in competition or training and the spikes do not all impact on an unmarked area or each within a mark. However, the behaviour of the blade with some of the spikes entering a pre-existing mark is closer to the behaviour with all spikes entering a pre-existing mark. Thus, in these configurations (spiked sole on TOB-2 or TOB-3) the first load data is disregarded and the mean value of the load on the other two tests is used for the data analysis.

### Local strain analysis

**Figure 6:**
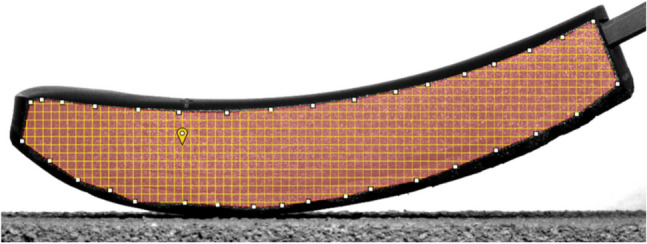
Initialisation of the Digital Image Correlation (DIC) procedure in VIC-2D. The subset grid appears in yellow on the working area (size of subset: 25 px).

To characterise the behaviour of the sole during the test, local strains on the speckled side of the sole are measured. They are computed by a DIC software (VIC-2D 6 from Correlated Solutions). This method uses camera images to measure the strain^27,28^. It tracks changes in patterns (rotation, translation and deformation) found in the image. Here the paint dots of the speckle in the image allows the strain to be recomposed. To find the different patterns, the working zone is sliced into small groups of pixels, called subsets (see Fig. 6). In our case, the mean size of subset (in pixels) used for treatment is 26 px (between 25 and 27 px) for running sole and 68.6 px (between 65 and 71 px) for spiked sole. Then the software computes the strains in the plane of the pattern in greyscale. For better precision in computation, the subset can overlap with a defined number of pixels. The resulting strain for each pixel is computed as the strain of each subset to which it belongs, weighted with a Gaussian weight. Here the overlap is 7 px for all sole. The tip of the sole shows a large displacement; thus, it is chosen to compute the strains incrementally: each image serves as a reference for the computation of the next. Then the strain is computed with a Lagrangian tensor.

## Detailed characterisation of prosthetic behaviour

### Global behaviour of the blade

In the following paragraphs, the ground–sole–blade interaction is assessed and discussed through apparent stiffness and dissipation. Lower energy dissipation and higher stiffness are sought to find settings for improving the behaviour of the prosthesis. The lower dissipation allows athletes to lose less energy in the prosthesis during the stride; all the energy stored during the compression of the blade is restored to the athlete at take-off. The higher stiffness sought by athletes allows them to store more potential energy in the blade during compression for an equivalent displacement amplitude.

#### Influence of load line alignment

**Figure 7:**
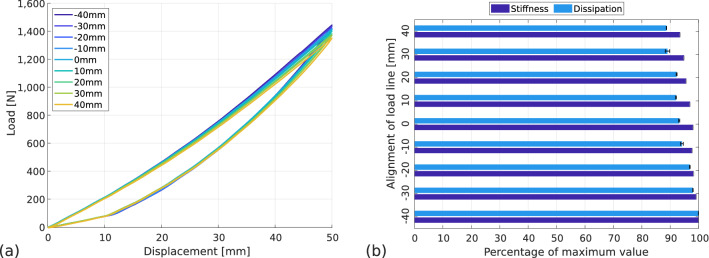
Influence of load line alignment on prosthetic behaviour. Flooring type: TOB-3, prosthesis-ground angle: horizontal, sole type: running. (**a**) Force–displacement curves, (**b**) normalised stiffness and dissipation with standard deviation.

To study the influence of load line on the RSPs’ behaviour, the blade is equipped with a running sole, the flooring is chosen rigid (TOB-3) and the prosthesis-ground angle flat ($\theta =$ 0$^\circ$). Then various load line positions are studied: − 40 mm, − 30 mm, − 20 mm, − 10 mm, 0 mm, 10 mm, 20 mm, 30 and 40 mm. The 0 mm position is the reference position (also called fitted position). The force–displacement curves are presented in Fig. 7a. There is no noticeable difference between the curves, but the maximum force reached decreases with increasing load line offset.

In Fig. 7b, the values of stiffness and dissipation are represented in proportion to the maximum value. For stiffness, the maximum value is 28.9 $\hbox {kN\,m}^{-1}$ and for dissipation, the maximum percentage is 19.9%. The maximum stiffness is reached for the − 40 mm position, the minimum stiffness is reached for the 40 mm position and is 6% lower. The maximum dissipation is also reached for the − 40 mm position, the minimum dissipation is also reached for the 40 mm position but is 11% lower. Between the two extrema, the values of stiffness and dissipation seem to decrease linearly.

This shows that the load line offset is not a critical parameter for athletes regarding performance, they can adjust the position of the blade tip to get a good feel for it without risking a loss of performance. However, to achieve fine optimisation and reduce dissipation while increasing stiffness, the position recommended by prosthetists is a good compromise.

#### Influence of prosthesis-ground angle

**Figure 8:**
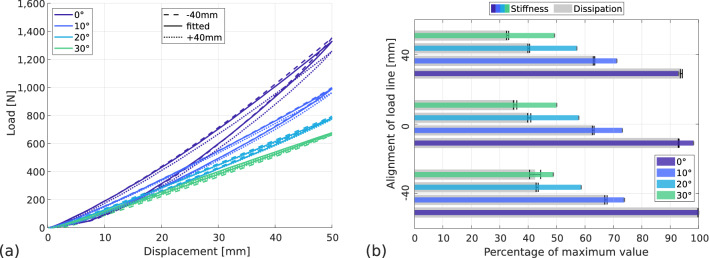
Influence of prosthesis-ground angle and alignment of load line on prosthetic behaviour. Flooring type: AT-WS, sole type: running. (**a**) Force–displacement curves, (**b**) normalised stiffness and dissipation with standard deviation.

To study the influence of prosthesis-ground angle on the RSPs’ behaviour, the blade is equipped with a running sole and the flooring is chosen as an athletics track (AT-WS). Then various prosthesis-ground angles are studied: $\theta =$ 0$^\circ$, 10$^\circ$, 20$^\circ$ and 30$^\circ$. To better see the influence of the prosthesis-ground angle, various alignments of the load line are also used: − 40 mm, the reference position and 40 mm. The force-displacement curves are presented in Fig. 8a. As mentioned before and for angles up to 20$^\circ$, the stiffness is higher for the − 40 mm position than for the 40 mm position and the adjusted position is in between. The prosthesis-ground angle has slightly more influence on the behaviour of the blade in the adjusted load line position than for the other alignments.

In Fig. 8b, the values of stiffness and dissipation are represented in proportion to the maximum value. For stiffness, the maximum value is 27.1 $\hbox {kN\,m}^{-1}$ and for dissipation, the maximum percentage is 13.5%. The maximum stiffness is reached for the − 40 mm position at $\theta =$ 0$^\circ$, the minimum stiffness is reached for the − 40 mm position at $\theta =$ 30$^\circ$ and is 51% lower. The maximum dissipation is also reached for the − 40 mm position at $\theta =$ 0$^\circ$, the minimum dissipation is reached for the 40 mm position at $\theta =$ 30$^\circ$ and is 67% lower. Adding an angle between the prosthesis and the ground decreases both the stiffness and the hysteretic dissipation. It shows that the stiffness of the blade changes through the stride, confirming the results of Beck et al.^21^.

Since the maximum stiffness for all load line alignments is achieved at the flat prosthesis-ground angle, this suggests that manufacturers use this prosthesis-ground angle only to optimise and evaluate the behaviour of their blades. However, adding an angle between the prosthesis and the ground still does not allow to find an optimal setting to have the lowest possible dissipation while maintaining high stiffness. But the difference in behaviour as a function of $\theta$ suggests a possibility for the sporting gesture: the lower stiffness for $\theta \ne$ 0$^\circ$ may offer greater ease of compression of the blade for the athlete at touchdown. Then, if take-off occurs with $\theta \approx$ 0$^\circ$, the greater apparent stiffness will create a gain in energy for the jumper. However, this is not the case in practice, the angle varies during the last stride. But it remains possible to work on the angle of touchdown and the angle of take-off in the light of these data to try to achieve optimum energy restitution.

#### Influence of sole type

**Figure 9:**
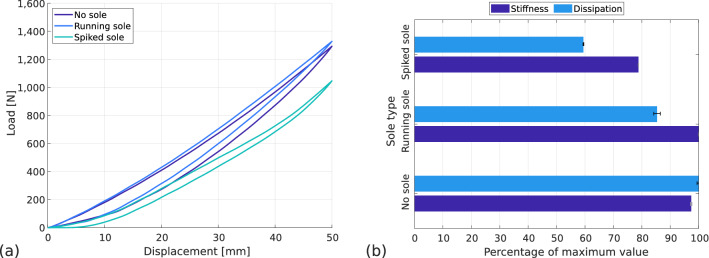
Influence of sole type on prosthetic behaviour. Flooring type: AT-WS, prosthesis-ground angle: horizontal, alignment: fitted. (**a**) Force–displacement curves, (**b**) normalised stiffness and dissipation with standard deviation.

To study the influence of sole type on the RSPs’ behaviour, the blade is tested at the fitted position on an athletic track (AT-WS) with a flat prosthesis-ground angle ($\theta =$ 0$^\circ$). Then various sole configurations are studied: spiked sole, running sole and no sole. The force–displacement curves are presented in Fig. 9a. Stiffness and dissipation values are similar between the running sole and no sole configuration, while the spiked sole shows lower stiffness and dissipation.

In Fig. 9b, the values of stiffness and dissipation are represented in proportion to the maximum value. For stiffness, the maximum value is 26.6 $\hbox {kN\,m}^{-1}$ and for dissipation, the maximum percentage is 14.6%. The maximum stiffness is reached with the running sole, the minimum stiffness is reached with the spiked sole and is 21% lower. The maximum dissipation is reached without sole, the minimum dissipation is reached with spiked sole and is 41% lower. The high dissipation in the case where the blade is compressed without a sole is due to the interaction with the athletic track: the carbon fibre of the blade is in direct contact with the ground and slips more than the rubber-like material of the running sole, dissipating more energy in sliding and friction.

**Figure 10:**
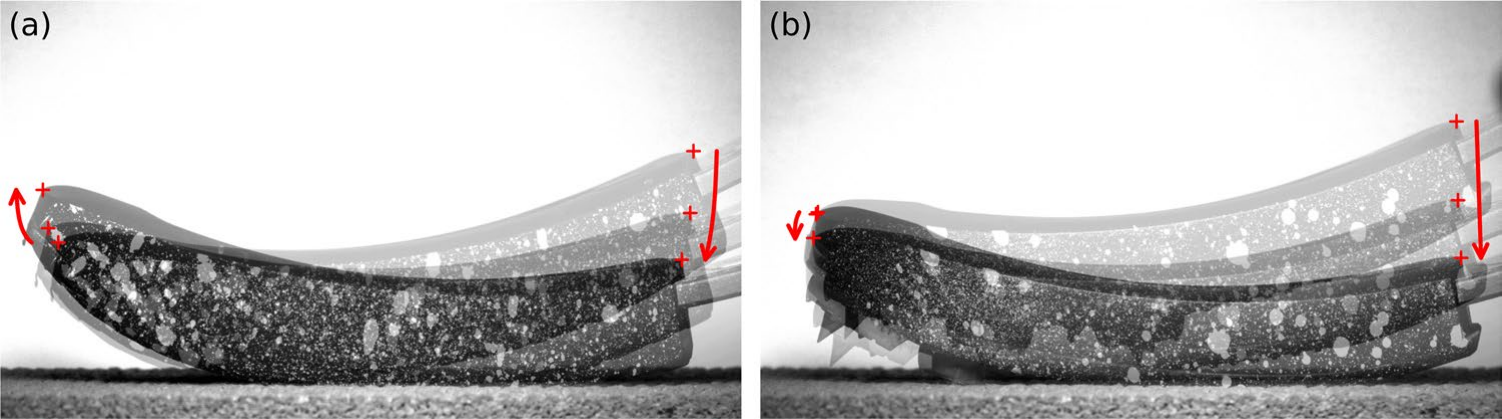
Kinematics of the blade tip during loading. Flooring type: AT-WS, prosthesis-ground angle: horizontal, alignment: fitted, sole type: (**a**) running, (**b**) spiked.

The difference in stiffness between the spiked sole and the running sole may be induced by the difference in the kinematics of the blade tip on the ground (see Fig. 10). With the spiked sole, there is no translation of the contact point during loading because the spikes indent in the flooring and prevent the contact point from moving. Thus, a part of the force applied on the ground is horizontal and is not recorded by the load cell of the testing machine which explains the lower stiffness in that case.

These results show the major role played by the soles on the behaviour of the prosthesis. The setup allows to quantify this impact and to better choose the soles among those available from the manufacturer, but without forgetting that athletics necessarily requires the use of spikes.

#### Influence of flooring type

**Figure 11:**
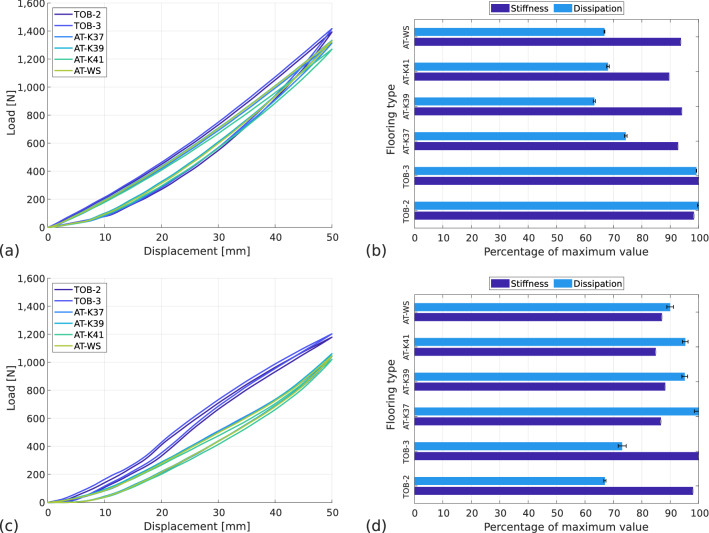
Influence of flooring type on prosthetic behaviour. Prosthesis-ground angle: horizontal, alignment: fitted, (**a**) sole type: running, force–displacement curves and (**b**) normalised stiffness and dissipation; (**c**) sole type: spiked, force–displacement curves and (**d**) normalised stiffness and dissipation with standard deviation.

To study the influence of flooring on the RSPs’ behaviour, the blade is tested at the fitted position with a flat prosthesis-ground angle ($\theta =$ 0$^\circ$). Then the six floorings are studied: the two take-off boards and the four athletics tracks presented previously. The tests are carried out twice, first with a running sole and then with a spiked sole. The force–displacement curves are presented in Fig. 11a for the running sole and in Fig. 11c for the spiked sole. Firstly, two groups of curves with different apparent stiffnesses appear for the spiked sole. The first one with take-off boards and the second one with athletics tracks. This difference is less important with the running sole but still appears. Secondly, the shape of the curve changes according to the sole used and the type of flooring. The evolution of forces for running sole is polynomial and similar on all floorings, while for spiked sole the behaviour is more complex with a different behaviour between athletics tracks and take-off boards.

In Fig. 11b,d, the values of stiffness and dissipation are represented in proportion to the maximum value. For running sole, the maximum stiffness value is 28.3 $\hbox {kN\,m}^{-1}$ and the maximum percentage of dissipation is 18.7%. The maximum stiffness is reached on TOB-3, the minimum stiffness is reached on AT-K41 and is 10% lower. The maximum dissipation is reached on TOB-2, the minimum dissipation is reached on AT-K39 and is 37% lower. For spiked sole, the maximum value of stiffness is 24.1 $\hbox {kN\,m}^{-1}$ and the maximum percentage of dissipation is 9.7%. The maximum stiffness is reached on TOB-3, the minimum stiffness is reached on AT-K41 and is 15% lower. The maximum dissipation is reached on AT-K37, the minimum dissipation is reached on TOB-2 and is 33% lower.

The difference in maximum stiffness observed between the two types of sole on take-off boards is explained by the kinematics of the blade tip seen earlier in Fig. 10: the contact point moves with the running sole while it does not with the spiked sole. This induces a loss due to more horizontal forces with the spiked sole on take-off boards.

A distinction in stiffness loss between take-off boards and athletics tracks with the two types of sole appears. The loss of stiffness is more important with spiked sole (12.5% on average) than with running sole (6.5% on average). This difference is explained by the difference in the behaviour of each sole on each type of flooring. For the running sole, the point of contact with each flooring moves, but the sliding is harder on the athletics tracks due to a higher coefficient of friction of the sole on the elastomer material of these surfaces than on the wood of the take-off board. For the spiked sole, there is no translation of the point of contact with any flooring because the spikes indent them, but athletics tracks are deformable, unlike take-off boards. The coefficient of friction of the running sole on the take-off boards has less influence than the blocking of the spiked sole on the loss of horizontal force, and thus on the stiffness.

A distinction in dissipation loss between take-off boards and athletics tracks with the two types of soles also appears but in a different direction. The running sole dissipates 31.5% less on average on athletics tracks than on take-off boards. The spiked sole dissipates 25% less on average on take-off boards than on athletics tracks. The lesser dissipation of spiked sole on take-off boards confirms the advantage in the long jump of performing the last stride on them. Not only does the athlete ensure that the full length of the jump is included in the performance measurement, but less energy is lost in the compression of the prosthesis. The setup makes it possible to quantify the energy gain as a function of the flooring, and optimisation according to the specific type of athletics track is also possible.

### Local behaviour of the sole

**Figure 12:**
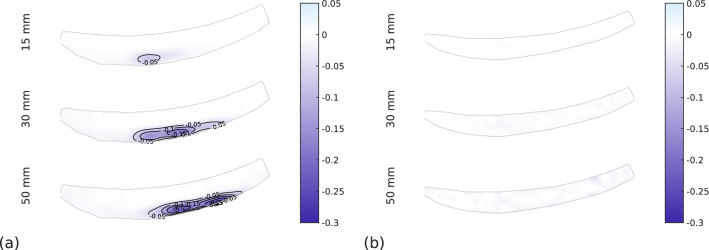
Sole strain profile as a function of displacement loading. Flooring type: AT-WS, prosthesis-ground angle: horizontal, alignment: fitted, sole type: (**a**) running, (**b**) spiked.

In the next paragraph, strains in the sole are discussed. Fig. 12 shows the evolution of the major principal strain in the soles during loading at a flat prosthesis-ground angle with the fitted alignment of the load line and on AT-WS athletics track. The principal strain is considered to be the vertical strain since the sole is used in compression. On the running sole (Fig. 12a), the strain value increases with the displacement and the localisation of its maximum zone moves with the translation of the contact point between the sole and the ground. On spiked sole (Fig. 12b), there is no strain localisation and the maximum value is lower than for running sole. This difference is explained by the presence of a rigid polymer part on the underside of the spiked sole to accommodate the spikes. This part stiffens the sole and distributes the forces over the entire underside of the sole. In contrast, in the running sole, the elastomer material that makes up the sole is in direct contact with the ground and does not distribute the forces.

**Figure 13:**
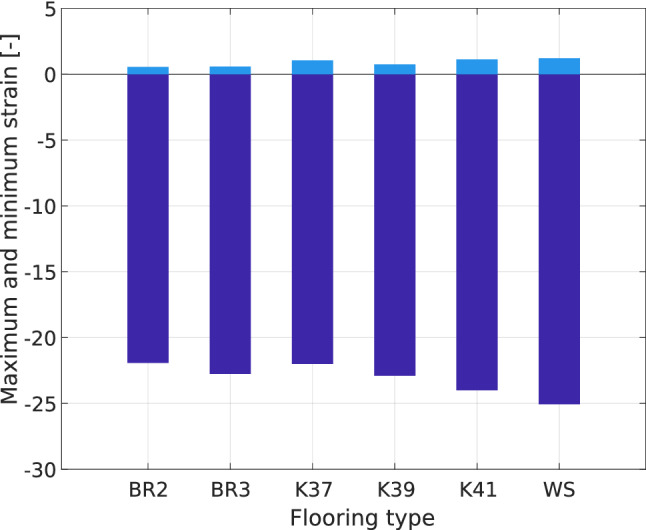
Principal compression (in dark blue) and tensile (in light blue) strain values as a function of the flooring type. Prosthesis-ground angle: horizontal, alignment: fitted, sole type: running.

Figure 13 represents the maximum value of the principal strain in running sole at a flat prosthesis-ground angle with the fitted alignment of load line on different floorings. The value is computed independently of the location of this maximum. The maximum tensile strains are always lower than the compression strains. Such tensile strains in the sole are due to the Poisson effect in the material since sole materials are rubber-like materials. There is no significant difference in the value of the compressive strain between the different floorings.

## Discussion on the protocols and results

### Consistency of the repeated loading-unloading cycles

To ensure consistency, the tests were repeated three times in a row with the same parameters, and with the same contact point between the prosthesis and the ground. A statistical analysis cannot be carried out with so few tests, but it is important to note that for a given condition, the tests are repeatable with very small deviations in terms of stiffness or dissipated energy. The estimates of the standard deviations give very low values, as shown in the set of results presented. A larger database will be needed to better assess the statistical dispersion, but the repeatability of the experimental set-up can be considered as guaranteed.

### Instantaneous stiffness of the spiked sole

The behaviour of the blade when using the spiked sole is more complex than when using the running sole (see Fig. 11). To study this behaviour, instantaneous stiffness has been defined in a similar way to effective stiffness. It is computed as the value of the force at a given time (*F*(*t*)) divided by the displacement at that time (*U*(*t*)):

$$\begin{aligned} k^*(t_i) = \displaystyle \frac{F(t_i)}{U(t_i)} \end{aligned}$$

**Figure 14:**
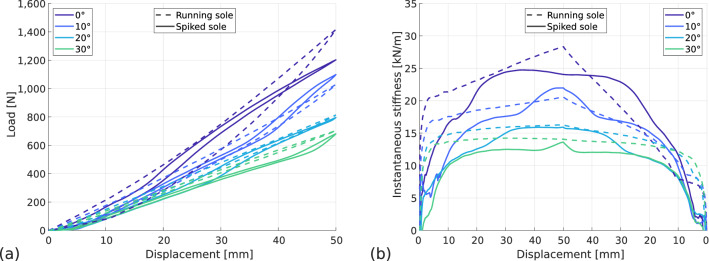
Influence of prosthesis-ground angle and sole type on prosthesis behaviour. Flooring type: TOB-3, alignment: fitted. (**a**) Force–displacement curves, (**b**) instantaneous stiffness.

Figure 14 shows the evolution of the instantaneous stiffness for different prosthesis-ground angles and different sole types. The influence of the prosthesis-ground angle is noticeable, as mentioned previously, on the value of the apparent stiffness: it decreases with the increase of the angle. However, for running sole the instantaneous stiffness evolves in an almost linear way beyond a certain displacement threshold. This threshold of displacement (in load as well as in unloading) is due to a possible lack of full contact at the beginning and the end of the test between the sole and the flooring. While increasing the prosthesis-ground angle, the instantaneous stiffness becomes almost constant for the running sole. For the spiked sole, the evolution is more complex with variations in stiffness rate. These increases do not occur at the same time depending on the angle of the ground, they appear later as the angle increases.

This behaviour for configurations with the spiked sole is probably due to the presence of the spikes. On the spiked sole, there are six spikes as mentioned above; these spikes are distributed two by two in the front, middle and rear of the sole. Depending on the prosthesis-ground angle, the number and position of spikes in contact with the ground change. This can explain the difference of stiffness variation rate during the tests.

Moreover, this behaviour can impact the way the athlete feels the reaction of the prosthesis during a stride, but these variations are less important than those due to the angle of the ground. They are therefore likely to be confused, in the athlete’s perception, with the change in prosthesis-ground angle during the contact phase.

Also, as mentioned before, the spiked sole dissipates more energy than the running sole. But even if the running sole dissipates more, its stiffness stays higher. Then it would be possible, by modifying the spiked sole material, to dissipate more without losing stiffness. This could protect the residual limb from the shocks caused by each step and help to prevent the appearance of injuries for athletes.

### Protocol improvements

**Figure 15:**
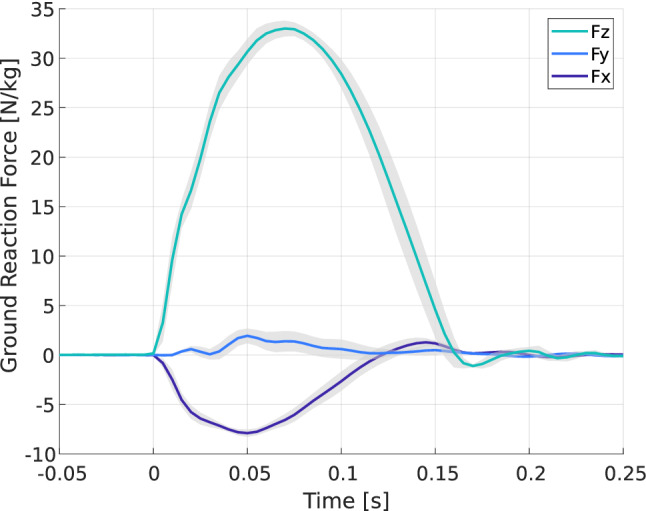
Ground reaction forces (GRFs) measured at take-off for a half-distance run-up. The forces are approximately twice smaller than for a full-distance run-up. Full lines: mean values and grey areas: standard deviations.

The actual Ground Reaction Forces (GRFs) that occur in running and jumping are complex (see Fig. 15). They include multidirectional reactions and the resultant force varies in direction during the stride^29^. However, in long jump, particularly the take-off, the vertical reaction prevails. For a full run-up distance, the force in this direction is approximately six times the body weight^29,30^ and four times bigger than antero-posterior forces. By consequence, measuring the efforts only in the vertical direction allows to estimate a significant part of the prosthesis reaction. Furthermore, by changing the prosthesis-ground angle, part of this multidirectional reaction is mimicked. When the ground is inclined, a part of the vertical forces measured is comparable to the antero-posterior forces of the last impulse.

Once these forces have been characterised for a given athlete and a given type of blade (as shown in Fig. 15), it would be relevant to establish an experimental base by controlling the loading in terms of force rather than displacement. It would then be possible to get as close as possible to the athlete’s real solicitation by guaranteeing that the maximum effort would not cause the blade to break. Although the results presented in the previous sections can be partly extrapolated for a force control, it would be interesting to make comparisons at a given maximum effort. The measurement of the energy dissipated would then be more representative of reality and would make it possible to adapt the sporting gesture and the equipment more effectively.

Another perspective for the protocol concerns the compression speed used: they are much smaller than real blade deformation speed during a stride. But this study intends to understand better the behaviour of the prosthesis depending on different parameters. The setup is designed to make the parameters independent and evaluate their influence individually. Only the slow speed made it possible. For the future, we plan to create a fully dynamic setup to test the prosthesis and exploit the results presented here to select the useful experiment parameters.

A final possible improvement of the setup involves the prosthesis-ground angle. During the last stride, this angle varies with time while in this study it is fixed. To improve the setup, it would be possible to motorise the angle mounting part. This is one of the perspectives envisaged for the continuation of this study.

## Conclusion and outlook

In conclusion, the developed innovative setup and method allow the mechanical behaviour of tibial RSPs and the local behaviour of soles to be characterised. The setup is a simple experiment based on a testing machine and a camera. It assesses two indicators relevant to coaches and athletes in athletics: secant stiffness and energy dissipation. Both may have influence on jumping performance and the risk of injury. The setup is basic enough to be mounted on most of the testing machines with few adaptations. The test parameters, the boundary conditions and the loading conditions are well controlled. All parameters vary in ranges that are close to the ones used in athletics (materials, values, etc.), making the setup a valuable tool to understand and adjust the blade behaviour without involving athletes and risking injuring them.

The four parameters assessed are the load line offset, the prosthesis-ground angle, the sole type and the flooring type. We find that some of these parameters have more influence than others. The load line alignment has little to no influence on the behaviour of the ground-sole-blade interaction. The influence of the flooring type depends on the sole used. The interesting result here is that with the spiked sole on a take-off board there is less energy dissipation. This reinforces for the athletes the relevance of having the last stride on the take-off board to gain both distance and stiffness. The prosthesis-ground angle influences the stiffness: an increase in the angle brings a significant decrease in stiffness. It suggests that it may be possible to find a touchdown and take-off angle that optimises the stiffness of the prosthesis from the athlete’s point of view. To improve the performance, it is interesting to find a touchdown angle that allows deforming the blade easily and a take-off angle that maximises the stiffness. The type of sole affects the kinematics of the blade tip in interaction with the ground. However, this effect is harder to use for athletics since the practice of sport imposes the use of spikes. One issue that could be studied further is the position and number of spikes on the sole.

The camera images enable following the strain of the sole through the compression process. The mapping of strain gives an idea of how the stride unfolds for athletes equipped with a running sole. This suggests that it can be possible to improve the sole to distribute the strain or change the localisation of its maximum. The principal compression as a function of the flooring type can also help in the future to find flooring better fitted for para-athletics.

In future work, it might also be interesting to test other blade shapes and stiffnesses and other soles. A sensitivity study would help athletes to choose the best RSP for long jumping.

## Data Availability

The datasets used and/or analysed during the current study available from the corresponding author on reasonable request.
